# Significance of Malignant Peritoneal Cytology on the Survival of Women with Early-Stage Cervical Cancer: A Japanese Gynecologic Oncology Group Study

**DOI:** 10.3390/jcm8111822

**Published:** 2019-11-01

**Authors:** Koji Matsuo, Muneaki Shimada, Shinya Matsuzaki, Hiroko Machida, Yoshikazu Nagase, Toshiaki Saito, Shoji Kamiura, Takashi Iwata, Toru Sugiyama, Mikio Mikami

**Affiliations:** 1 Division of Gynecologic Oncology, Department of Obstetrics and Gynecology, University of Southern California, Los Angeles, CA 90033, USA; zacky_s@gyne.med.osaka-u.ac.jp; 2 Norris Comprehensive Cancer Center, University of Southern California, Los Angeles, CA 90089, USA; 3 Department of Obstetrics and Gynecology, Tottori University, Tottori 683-8504, Japan; 4 Department of Obstetrics and Gynecology, Tohoku University, Miyagi 980-8574, Japan; 5 Department of Obstetrics and Gynecology, Tokai University, Kanagawa 259-1193, Japan; hiroko.machida@tokai.ac.jp (H.M.); mmikami@is.icc.u-tokai.ac.jp (M.M.); 6 Department of Obstetrics and Gynecology, Osaka University, Osaka 565-0871, Japan; doctoryoshikazu@gmail.com; 7 Gynecology Service, National Hospital Organization Kyushu Cancer Center, Fukuoka 811-1395, Japan; saito.toshiaki.hf@mail.hosp.go.jp; 8 Department of Gynecologic Oncology, Osaka International Cancer Institute, Osaka 541-8567, Japan; kamiura-sh@mc.pref.osaka.jp; 9 Department of Obstetrics and Gynecology, Keio University School of Medicine, Tokyo 160-8582, Japan; iwatatakashi@1995.jukuin.keio.ac.jp; 10 Department of Obstetrics and Gynecology, Iwate Medical University, Iwate 020-8505, Japan; sugiyamatoru0802@yahoo.co.jp

**Keywords:** cervical cancer, radical hysterectomy, peritoneal cytology: malignant cytology, adjuvant therapy, chemotherapy, survival

## Abstract

This study examined the association between peritoneal cytology and survival in early-stage cervical cancer. This is a nationwide multicenter retrospective study, examining consecutive women with clinical stage IB1-IIB cervical cancer who underwent radical hysterectomy with available peritoneal cytology results from 2004–2008. Propensity score inverse probability of treatment weighting was used to assess the impact of malignant peritoneal cytology on survival. Among 1409 analyzed cases, 88 (6.2%) had malignant peritoneal cytology. On weighted models, malignant peritoneal cytology was associated with decreased disease-free survival (hazard ratio (HR) 1.78, 95% confidence interval (CI) 1.36–2.32) and overall survival (OS, HR 1.93, 95% CI 1.44–2.59). On sensitivity analyses, malignant peritoneal cytology was associated with decreased OS in adenocarcinoma/adenosquamous carcinoma, high-risk early-stage disease and those who received concurrent chemo-radiotherapy. However, among women who received postoperative systemic chemotherapy, malignant peritoneal cytology was not associated with OS (HR 1.21, 95% CI 0.72–2.04). A systematic review, including our results, showed that malignant peritoneal cytology was associated with decreased OS (HR 4.03, 95% CI 1.81–8.99) and increased recurrence in squamous carcinoma (odds ratio 1.89, 95% CI 1.05–3.39) and adenocarcinoma (odds ratio 4.30, 95% CI 2.30–8.02). In conclusion, the presence of malignant cells in peritoneal cytology is associated with decreased survival in early-stage cervical cancer. The possible benefit of systemic chemotherapy in this subgroup merits further investigation.

## 1. Introduction

Cervical cancer is commonly associated with persistent oncogenic human papillomavirus (HPV) infection. Globally, cervical cancer is the most common female malignancy, with nearly 527,600 newly diagnosed women in 2012 [1]. Treatment for cervical cancer is largely based on the cancer stage. The radical hysterectomy-based approach is commonly utilized for early-stage cervical cancer, and surgical-pathological information obtained from the hysterectomy specimen is valuable to identify factors which place the patient at increased risk of recurrence or death [2]. Of these, the high risk factors recognized in the current guidelines are pelvic nodal metastasis, parametrial tumor involvement and positive surgical margins, while those recognized as the intermediate-risk factors are deep stromal tumor invasion, large tumor size and lympho-vascular space invasion (LVSI) [2,3,4]. 

There has long been an interest in determining if the presence of malignant cells in the peritoneal cytology is a possible surgical-pathological risk factor in cervical cancer [5]; however, the prognostic significance in women with early-stage cervical cancer has not been well studied [6,7,8,9,10]. Overall, these prior studies have been limited by small sample size, examination of only certain histology types, or have grouped advanced-stage disease together with early-stage disease, leaving the findings difficult to interpret, specifically in the setting of early-stage disease [6,7,8,9,10,11,12,13,14,15,16].

Moreover, the treatment implication of malignant cells in the peritoneal cytology testing remains understudied. The current standard for the postoperative therapy for cervical cancer with identified risk factors is pelvic radiation [2]. This would not be anticipated to affect disease outside of the pelvis. In the setting of malignant peritoneal cytology, systemic therapy with chemotherapy might be more effective than therapy limited to the pelvis, as the malignant cells can spread beyond the bounds of this anatomically described space targeted with pelvic irradiation [17]. To this point, there has been no study which has examined the utility of adjuvant therapy for cervical cancer with malignant peritoneal cytology.

The objective of the study was to examine the association between peritoneal cytology test results and the survival of women with early-stage cervical cancer. Outcomes associated with different types of adjuvant therapy were further assessed. 

## 2. Materials and Methods

### 2.1. Data Source

This is a secondary analysis of a prior nationwide multicenter retrospective study conducted in Japanese Gynecologic Oncology Group (JGOG)-designated institutions [18,19,20,21,22,23,24,25]. The study concept and participation call for this landmark collaborator study was initially announced to all JGOG-designated institutions (182 sites), and 116 (63.7%) of these sites voluntarily participated in this study (JGOG-1072S). Institutional Review Board approval was obtained at Tottori University, (National University Corporation Tottori University, Tottori, Empire of Japan), which served as the host institution, and participating JGOG-participating institutions obtained their own approvals at each site as indicated. 

Detail of the utilized methodology was previously described [18,19,20,21,22,23,24,25]. Briefly, data collection took place at each study site by deploying a universal data entry form for the collection of clinical, tumor, treatment and survival information from archived medical records. Upon the completion of data collection by clinicians participating in the study, the anonymous de-identified data sheet was transferred to the host institution. The study period for the data acquisition was from 10/2012–2/2013. 

### 2.2. Eligibility Criteria

Consecutive women with clinical stage IB1-IIB cervical cancer who underwent radical hysterectomy and pelvic lymphadenectomy with available peritoneal cytology results from 1/2004–12/2008 were eligible for analysis. Cases with unknown peritoneal cytology results were excluded from the analysis.

### 2.3. Clinical Information

Clinical demographics included patient age, calendar year of surgery and clinical stage per the 2014 International Federation of Gynecology and Obstetrics (FIGO) system (stage IB1, IB2, IIA, and IIB) [26]. Surgical-pathological factors were grouped as: Histologic subtype (squamous cell carcinoma, adenocarcinoma, adenosquamous, and others), tumor size (>4 versus ≤4 cm), parametrial tumor involvement (yes versus no), deep stromal invasion (outer half versus inner half), LVSI (yes versus no), uterine corpus tumor invasion (yes versus no), malignant cells in peritoneal cytology (yes versus no) and ovarian metastasis (yes versus no). 

Lymph node status included performance of lymphadenectomy and metastasis (yes versus no) in both the pelvic and para-aortic chains. In cases in which lymphadenectomy was performed, the number of sampled lymph nodes was collected. Hospital surgical volume per site was grouped as low, mid and high. Treatment type included the use of neoadjuvant chemotherapy (yes versus no) and adjuvant therapy (concurrent chemoradiotherapy (CCRT), radiotherapy alone, and systemic chemotherapy). Survival outcomes included follow-up, presence of recurrence, vital status and cause of death. 

### 2.4. Study Definition

Cutoff and grouping for the clinico-pathological variables were based on previous studies [18,19,20,21,22,23,24,25]. Definition of hospital surgical volume was based on a prior study: <32 cases for low-volume, 32–104 cases for mid-volume, and ≥105 cases for high-volume over five years [21]. High-risk early-stage cervical cancer was defined as the presence of parametrial tumoral invasion or nodal metastasis in this study [3]. Disease-free survival (DFS) was defined as the time interval between surgery and the first recurrence or death from disease. Overall survival (OS) was defined as the time interval between surgery and death (all-cause). Cases were censored at the last follow-up if there was no survival event as above. 

### 2.5. Statistical Consideration

The first-level analysis examined the demographic factors associated with the utilization of peritoneal cytology testing at the time of radical hysterectomy as a whole cohort. A recursive partitioning analysis was performed to construct a regression-tree model for clinico-pathological demographic patterns of the utilization of peritoneal cytology testing at the time of radical hysterectomy [27]. All preoperative factors (age, year, clinical stage, histology type, hospital surgical volume and neoadjuvant chemotherapy) were entered into the final model, and the chi-square automatic interaction detector method was used to determine the nodes with stopping rule at the levels of three. Among the determined nodes in this analysis, the incidence of peritoneal cytology testing was calculated.

The second-level analysis examined the independent factors and prognostic impact associated with malignant peritoneal cytology among the tested cases for peritoneal cytology. A binary logistic regression model with conditional backward methods was fitted to identify the independent clinico-pathological factors for malignant peritoneal cytology results, expressed with odds ratio (OR) and 95% confidence interval (CI). 

For survival analysis, the propensity score inverse probability of treatment weighting (PS-IPTW) was performed to corroborate the baseline differences between the two groups [28]. The PS-IPTW model creates a weighted cohort that differed based on treatment type (malignant versus negative cytology), but was similar with respect to other baseline demographics. 

First, the PS was estimated by fitting a multivariable binary logistic regression model to predict the malignant peritoneal cytology [29]. All the study variables except for clinical stage were entered into the model. The PS-IPTW approach assigned to malignant peritoneal cytology a weight of 1/PS, and to negative peritoneal cytology a weight of 1/(1-PS). Stabilized weight was used for analysis, and the threshold technique was used at the 1st and 99th percentile of the weight distribution [28]. In the weighted model, characteristics of the two groups were assessed for balance with a standardized difference (cutoffs: 0.2 for small, 0.5 for medium, and 0.8 for large size effect) [30,31].

On the PS-IPTW model, the Kaplan-Meier method was used to construct survival curves, and Cox proportional hazard regression models were fitted to estimate the hazard ratio (HR) with a 95% CI for malignant peritoneal cytology compared to negative peritoneal cytology. 

Various sensitivity analyses were undertaken to examine the robustness of the study results. First, the histology-specific impact of malignant peritoneal cytology was assessed (squamous and adenocarcinoma/adenosquamous) as prior studies predominantly focused on histology-specific effects. Second, the impact of malignant peritoneal cytology was tested in the high-risk early-stage cervical cancer, as there was medium effect size between the two groups in the PS-IPTW models. Third, the association between malignant peritoneal cytology and survival was examined based on adjuvant therapy type (CCRT, radiotherapy alone and systemic chemotherapy). PS-IPTW models were constructed in each subgroup. 

All statistical analyses were based on two-sided hypothesis and a *P* < 0.05 was considered statistically significant. Statistical Package for Social Sciences (version 24.0, Armonk, NY, USA) and R version 3.6.0 (R Foundation for Statistical Computing, Vienna, Austria) were used for all the analyses. The STROBE guidelines were consulted to outline this observational cohort study.

### 2.6. Systematic Review and Meta-Analysis

#### 2.6.1. Aim

The study team conducted a systematic literature review and meta-analysis to determine the impact of malignant peritoneal cytology on prognosis in early-stage cervical cancer. The primary focus was survival outcome, comparing malignant to negative peritoneal cytology results.

#### 2.6.2. Article Retrieval

A systematic literature search was executed on 7/31/2019 (31st of July, 2019), using multiple public searching engines (PubMed, Scopus and Cochrane Central Register of Controlled Trials (CENTRAL)). This systematic review followed the PRISMA guidelines [32]. Two investigators (SM and YN) independently performed the study selection (screening of titles, abstracts and full texts of relevant articles), as previously described [33]. 

Only English literature was included, and the search strategy involved the use of the following keywords [17]: These were “cervical cancer or carcinoma or malignancy or neoplasm”, “uterine cervical cancer or carcinoma or malignancy or neoplasm”, “squamous cell carcinoma of the cervix”, “adenocarcinoma of the cervix”, “cancer or carcinoma or invasive carcinoma of the cervix”, “cancer or carcinoma or invasive carcinoma of the uterine cervix”, “radical hysterectomy”, and “peritoneal cytology or peritoneal washing cytology or pelvic cytology or abnormal cytology or malignant cytology”. References of the identified articles were also reviewed, and articles that met the inclusion criteria were included.

#### 2.6.3. Inclusion and Exclusion Criteria

The inclusion criteria were based on the PICOS design [34]: (i) Patients with cervical cancer; (ii) response outcomes of performed intraoperative peritoneal cytology as intervention; (iii) there was no comparator; (iv) effect size for outcome; and (v) original articles with study design such as retrospective or prospective cohort studies, population-based case-control studies and randomized controlled trials. The exclusion criteria were as follows: (i) Insufficient survival or recurrence information; (ii) not in the field of interest; (iii) inclusion of advanced stage or recurrent cases; (iv) lack of a negative peritoneal cytology group, and (v) articles with case reports, case series, and systematic review.

#### 2.6.4. Meta-Analysis Plan 

From the eligible study data, survival outcome estimates for malignant versus negative peritoneal cytology were computed by utilizing the reported values’ 95% CI to estimate HR for OS, and the reported frequency number for recurrence to estimate OR for recurrence, respectively. Heterogeneity across studies was examined using I^2^, which measures the percentage of the total variation across studies [35]. Meta-analysis and the production of all graphics were performed using the Cochrane Collaboration’s RevMan 5.3 software [36]. For consistency, data from all outcomes (continuous and bivariate) were entered into RevMan 5.3 in such a way that negative effect sizes or relative risks less than one favored the active intervention. 

## 3. Results

### 3.1. Utilization of Peritoneal Cytology Testing

**Figure 1:**
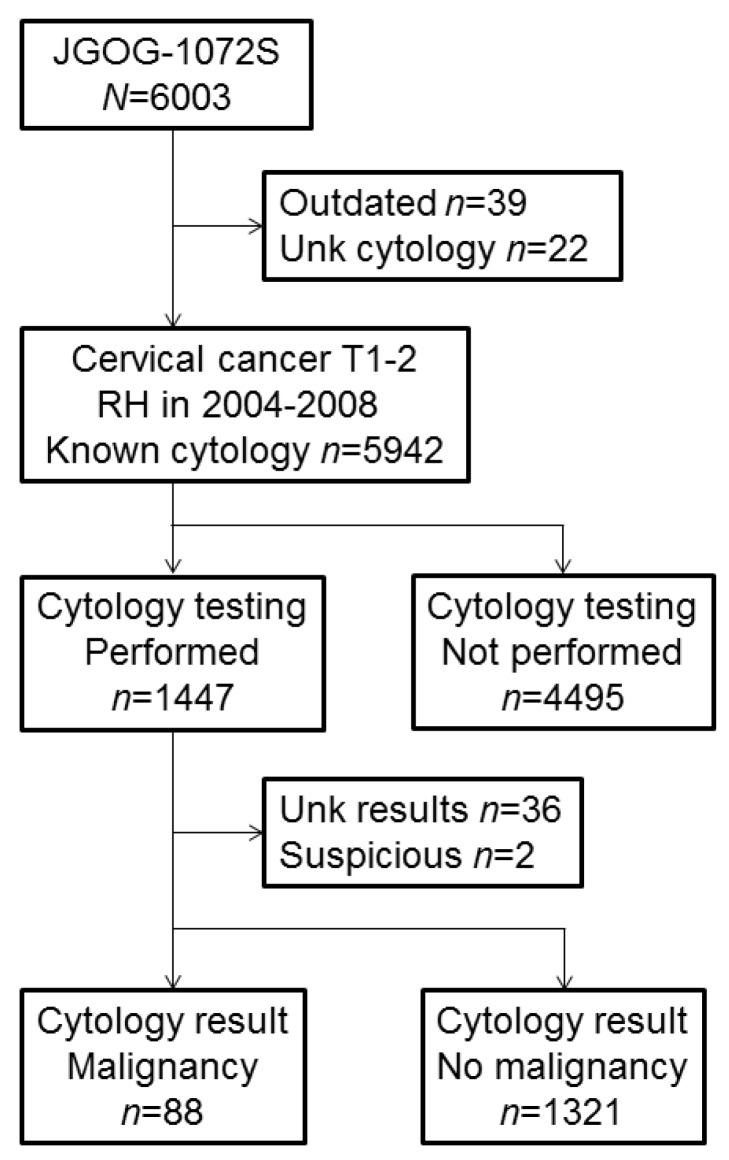
CONSORT diagram for study selection. Abbreviations: RH, radical hysterectomy; and unk, unknown.

**Figure 2:**
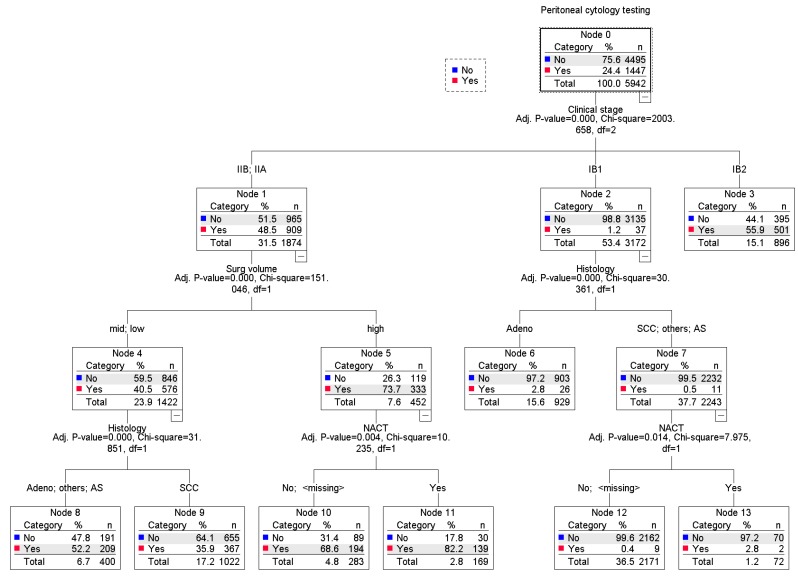
A regression-tree model for the utilization of peritoneal cytology testing at surgery. Abbreviations: SCC, squamous cell carcinoma; Adeno, adenocarcinoma; AS, adenosquamous; and NACT, neoadjuvant chemotherapy.

Among 5942 cases with known peritoneal cytology results, peritoneal cytology testing was utilized in 1447 (24.4%) cases (Figure 1). Clinico-pathological characteristics were largely different between the peritoneal cytology testing group and the non-testing group (Table 1), and there were eight unique patterns of the utilization identified in a regression-tree model (Figure 2). Of the identified factors, clinical stage was the strongest discriminatory factor for peritoneal cytology use (*P* < 0.001). More specifically, women with stage IIA-B disease who had surgery following neoadjuvant chemotherapy at a high surgical volume center had the highest utilization of peritoneal cytology testing (82.2%). In women who underwent primary surgery for stage IB1 disease with non-adenocarcinoma histology, the utility of peritoneal cytology was as low as 0.5% (absolute difference 81.7%). 

### 3.2. Risk Factors for Malignant Peritoneal Cytology

**Figure 3:**
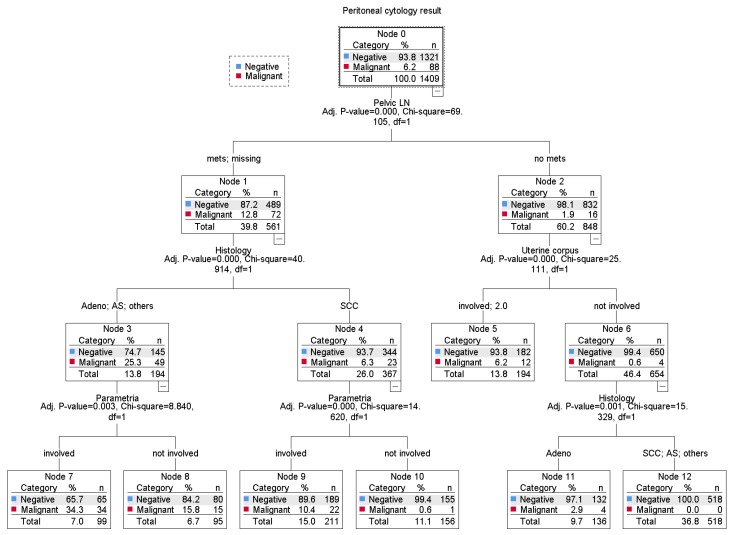
A regression-tree model for the malignant peritoneal cytology. Abbreviations: SCC, squamous cell carcinoma; Adeno, adenocarcinoma; and AS, adenosquamous.

Among 1409 cases with peritoneal cytology results either as malignant or negative, 88 (6.2%, 95% CI 5.0–7.5) had malignant cells in the peritoneal cytology, leaving 1321 (93.8%) who did not (Figure 1). On multivariable analysis (Table 2), there were five independent factors identified for malignant peritoneal cytology: Adenocarcinoma (OR 6.18, 95% CI 3.49–10.9), adenosquamous carcinoma (OR 2.57, 95% CI 1.08–6.13), pelvic lymph node metastasis (OR 6.51, 95% CI 3.43–12.3), parametrial involvement (OR 1.87, 95% CI 1.07–3.27), uterine corpus invasion (OR 2.74, 95% CI 1.61–4.67), and ovarian metastasis (OR 5.72, 95% CI 2.34–14.0). On a regression-tree model (Figure 3), there were seven unique patterns identified, of which non-squamous tumors with pelvic nodal and parametrial tumor involvement possessed a disproportionally high incidence of malignant peritoneal cytology (34.3%). 

### 3.3. Prognostic Significance of Malignant Peritoneal Cytology

**Figure 4:**
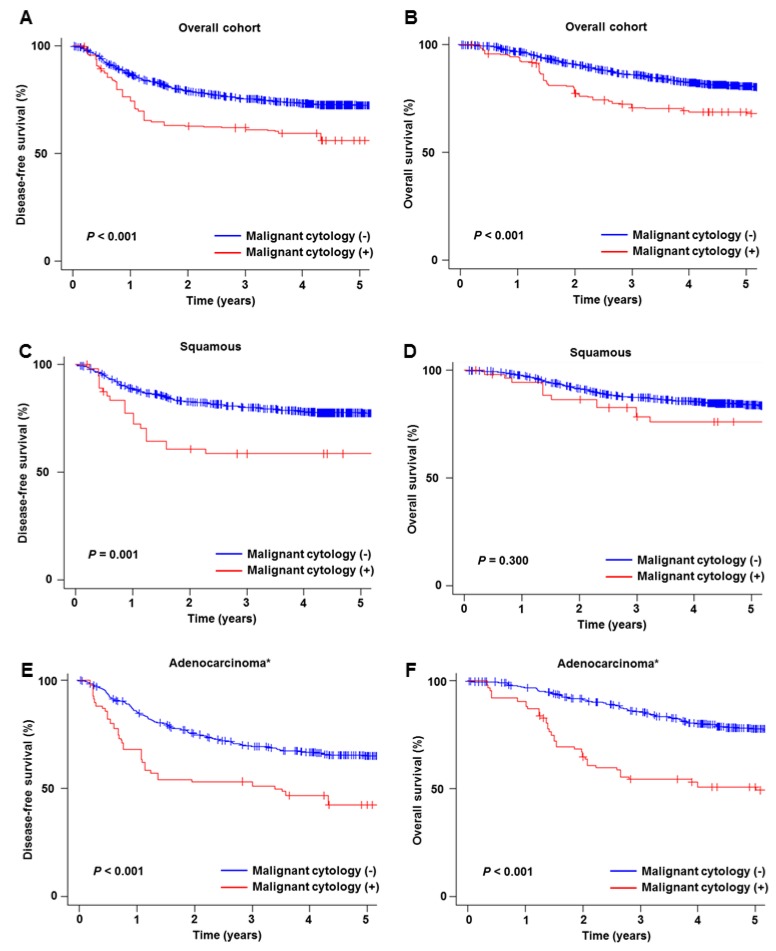
Survival outcome based on peritoneal cytology results. Kaplan-Meier curves are shown based on presence and absence of malignant cells in the peritoneal cytology tests for overall cohort (DFS for panel **A**, and OS for panel **B**), squamous cell carcinoma (DFS for panel **C**, and OS for panel **D**), and adenocarcinoma/adenosquamous carcinoma (DFS for panel **E**, and OS for panel **F**). Log-rank test for *P*-Values on propensity score inverse provability of treatment weighting. * including adenosquamous carcinoma. Abbreviations: DFS, disease-free survival; and OS, overall survival.

After PS-IPTW, clinico-pathological demographics were much more balanced between the two groups compared to the pre-weighting model (Supplemental Figure S1). Only pelvic nodal metastasis and parametrial tumor invasion exhibited the medium size effect in the PS-IPTW model. A total of 1512 cases were assessed for survival analysis (malignant peritoneal cytology *n* = 170, and negative peritoneal cytology *n* = 1342) in the PS-IPTW model, and with a median follow-up period of 5.4 (IQR 4.3–6.8) years there were 459 recurrences and 321 deaths recorded. On weighted models, the presence of malignant cells on peritoneal cytology was associated with decreased DFS (HR 1.78, 95% CI 1.36–2.32, *P* < 0.001; Figure 4A) and OS (HR 1.93, 95% CI 1.44–2.59, *P* < 0.001; Figure 4B) compared to negative peritoneal cytology. 

### 3.4. Sensitivity Analysis

**Figure 5:**
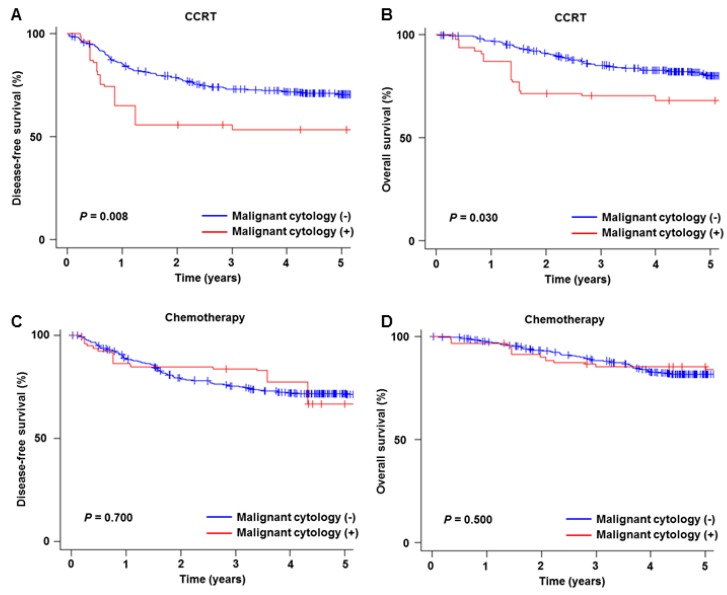
Survival outcome based on peritoneal cytology results per postoperative treatment. Kaplan-Meier curves are shown based on the presence and absence of malignant cells in the peritoneal cytology tests for CCRT cohort (DFS for panel **A**, and OS for panel **B**) and systemic chemotherapy cohort (DFS for panel **C**, and OS for panel **D**). Log-rank test for *P*-Values on propensity score inverse provability of treatment weighting. Abbreviations: CCRT, concurrent chemo-radiotherapy; DFS, disease-free survival; and OS, overall survival.

**Figure 6:**
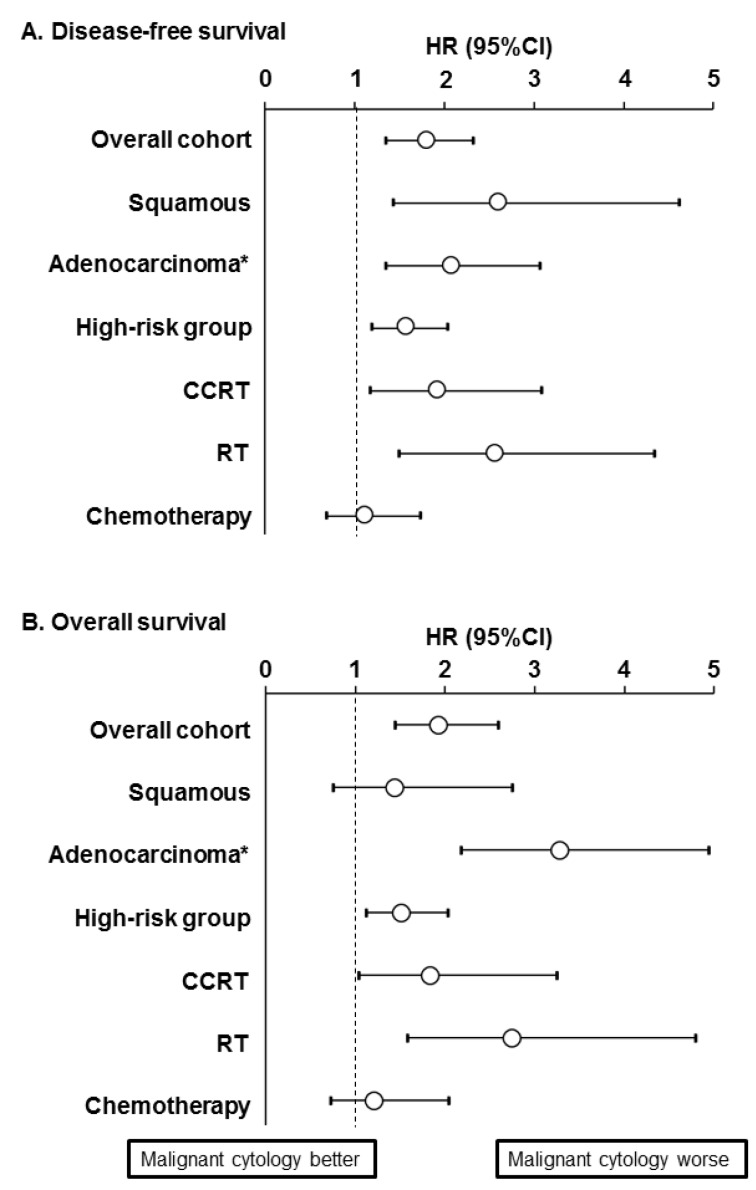
Forest plot for sensitivity analysis. HR for malignant peritoneal cytology is shown for (**A**) disease-free survival and (**B**) overall survival. All analyses were based upon propensity score inverse provability of treatment weighting (squamous carcinomas *n* = 956, adenocarcinomas/adenosquamous carcinomas *n* = 466, high-risk early-stage diseases *n* = 822, CCRT *n* = 404, RT alone *n* = 308, and systemic chemotherapy *n* = 424). Circles represent HR and bars represent 95%CI. * including adenosquamous carcinoma. High-risk group included pelvic lymph node metastasis and parametrial tumor involvement (any). Abbreviations: HR, hazard ratio; CI, confidence interval; CCRT, concurrent chemo-radiotherapy; and RT, radiotherapy.

Results of the sensitivity analysis are shown in Figure 4, Figure 5 and Figure 6. When histology type was stratified, malignant peritoneal cytology was significantly associated with decreased DFS in both squamous carcinoma (HR 2.58, 95% CI 1.44–4.62; Figure 4C) and adenocarcinoma/adenosquamous carcinoma (HR 2.05, 95% CI 1.36–3.07; Figure 4E) compared to negative peritoneal cytology. However, while adenocarcinoma/adenosquamous carcinoma was associated with decreased OS (HR 3.28, 95% CI 2.18–4.94; Figure 4F) squamous carcinoma was not (HR 1.44, 95% CI 0.75–2.75; Figure 4D). Among the cases with high-risk early-stage disease (Supplemental Figure S2), malignant peritoneal cytology was associated with decreased survival (HR for DFS 1.56, 95% CI 1.20–2.04; and HR for OS, 1.51, 95% CI 1.12–2.03).

Malignant peritoneal cytology was also associated with decreased survival among those who received CCRT (HR for DFS 1.90, 95% CI 1.17–3.08, Figure 5A; and HR for OS, 1.84, 95% CI 1.04–3.24, Figure 5B) and RT alone (HR for DFS 2.55, 95% CI 1.50–4.33; and HR for OS, 2.75, 95% CI 1.58–4.79). However, among women who received postoperative systemic chemotherapy, the presence of malignant cells was not associated with DFS (HR 1.09, 95% CI 0.68–1.73; Figure 5C) or OS (HR 1.21, 95% CI 0.72–2.04; Figure 5D). 

### 3.5. Systematic Review

#### 3.5.1. Overview

The search criteria are shown in Supplemental Figure S3. Out of a total of 1298 reviewed studies, 826 unique studies were identified. Of those, 39 studies were reviewed for abstract, and 29 studies were assessed for full-text review to assess the eligibility. Twenty-three studies describing advanced or recurrent cervical cancer, as well as studies that did not provide information on positive and negative results of peritoneal cytology were excluded. One systematic review article which examined the impact of malignant peritoneal cytology on prognosis in cervical cancer was also excluded [17]. 

Ultimately, the search identified five unique studies [6,7,8,9,10]. Together with the current JGOG study, six studies were assessed for survival outcome. For the JGOG study, the statistics were based on the weighted model value. Random-effect analysis was used to evaluate the HR for OS and OR of recurrence due to the moderate heterogeneity of studies (*I*^2^ > 50%; Supplemental Figure S4).

#### 3.5.2. All-Cause Mortality

**Figure 7:**
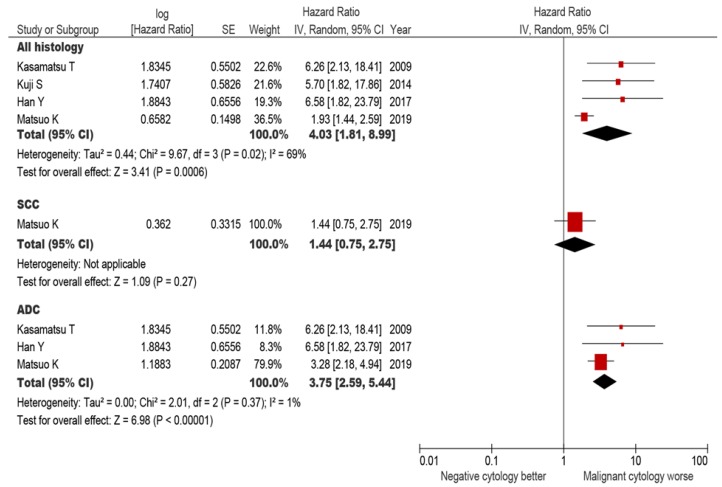
All-cause mortality for malignant peritoneal cytology (systematic review and meta-analysis). A forest plot from a random effects meta-analysis of six studies including our study stratified by inclusion criteria and ordered within stratum by year of publication and relative weight (%) of study. Centers of squares and horizontal bars through each indicate the point and 95% CI estimates of individual study odds ratio. Area of squares indicates the relative weights of the individual studies. Abbreviations: SCC, squamous cell carcinoma; and ADC, adenocarcinoma. Abbreviations: SCC, squamous cell carcinoma; and ADC, adenocarcinoma (including adenosquamous carcinoma in certain study).

The prognostic impact of malignant peritoneal cytology on OS was assessed in a total of 1983 patients from four studies (Figure 7). Irrespective to histology type, malignant peritoneal cytology was associated with a nearly four-fold increased risk of all-cause mortality when compared to negative peritoneal cytology (HR derived from multivariable analysis 4.03, 95% CI 1.81–8.99, *P* < 0.001). When stratified by histology type, only the JGOG study evaluated squamous histology. For adenocarcinoma histology, four studies reported the OS results, and malignant peritoneal cytology was associated with increased all-cause mortality compared to negative cytology (HR 3.75, 95% CI 2.59–5.44, *P* < 0.001). 

#### 3.5.3. Recurrence

**Figure 8:**
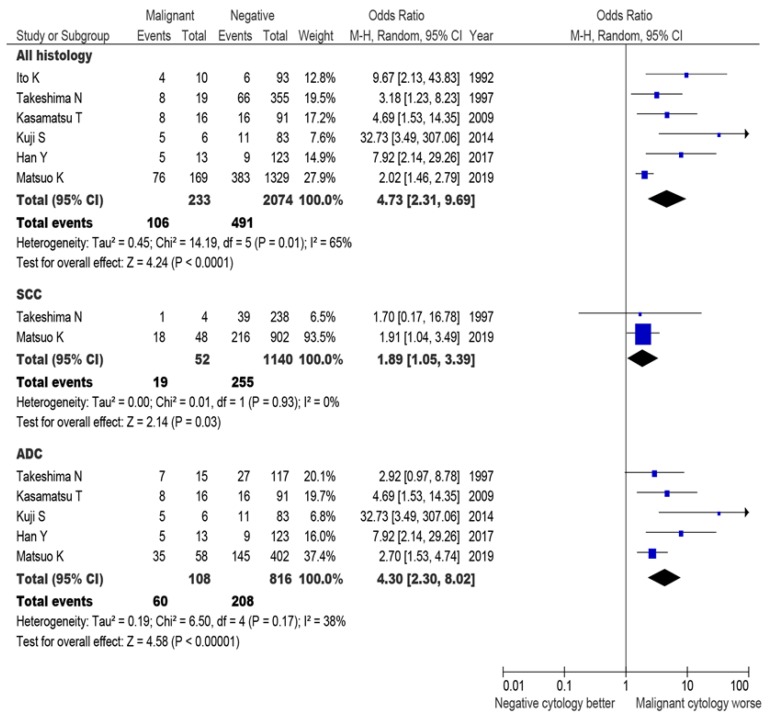
Recurrence risk for malignant peritoneal cytology (systematic review and meta-analysis). A forest plot from a random effects meta-analysis of six studies including our study stratified by inclusion criteria and ordered within stratum by year of publication and relative weight (%) of study. Centers of squares and horizontal bars through each indicate the point and 95% CI estimates of individual study odds ratio. Area of squares indicates the relative weights of the individual studies. Abbreviations: SCC, squamous cell carcinoma; and ADC, adenocarcinoma (including adenosquamous carcinoma in certain study).

A total of 2307 patients from six studies reported the results of cervical cancer recurrence (597 recurrences; Figure 8). In a pooled analysis, malignant peritoneal cytology was associated with increased recurrence risk (OR 4.73, 95% CI 2.31–9.69, *P* < 0.001). When stratified by histology types, two studies were available for squamous tumors (*n* = 1140), and five studies reported results for adenocarcinoma (*n* = 816). In both histology subtypes, malignant peritoneal cytology was associated with increased risks of recurrence: Squamous tumors OR 1.89 (95% CI 1.05–3.39, *P* = 0.030) and adenocarcinoma OR 4.30 (95% CI 2.30–8.02, *P* < 0.001). 

## 4. Discussion

### 4.1. Prognostic Significance

The current study validates the results of prior studies in that the presence of malignant cells in pelvic cytology testing at the time of radical hysterectomy is associated with decreased survival in women with early-stage cervical cancer. As prior studies were limited in the sample size (range, 105–448) and the number of malignant peritoneal cytology cases (range, 11–27), the findings from our study, which included a larger patient pool, are more reliable for interpretation of the analysis [6,7,8,9,10]. 

Moreover, our study is likely the first to show the prognostic impact of malignant peritoneal cytology in the setting of squamous histology. Previously, the squamous type was considered to have a low incidence of malignant peritoneal cytology, and has not been well studied [5]. Our team identified one study which examined the recurrence risk in squamous tumors, however survival analysis was not interpretable due to the small number of cases with malignant peritoneal cytology (*n* = 5) [9]. In our study, malignant peritoneal cytology was associated with an increased risk of recurrence of the squamous type. 

However, in contrast to adenocarcinoma/adenosquamous carcinoma types, malignant peritoneal cytology was not associated with decreased all-cause mortality in the squamous type. The exact causality of the findings is unknown, but it may be that the response to salvage therapy for recurrent tumors may differ between the squamous and adenocarcinoma types. It is noted that squamous tumors have a lower chemotherapy response compared to adenocarcinoma, and this difference may possibly explain our results [37]. 

### 4.2. Association for Historical Tumor Factors

Our study demonstrated that malignant peritoneal cytology was associated with aggressive tumor characteristics such as pelvic nodal metastasis, parametrial and uterine corpus tumor invasion and ovarian metastasis. This finding partly supports a recent meta-analysis that demonstrated an association between malignant peritoneal cytology and an increased risk of pelvic lymph node metastasis [17]. 

In our study, when adenocarcinoma/adenosquamous carcinoma spreads to parametria and pelvic nodes, the risk of malignant peritoneal cytology was disproportionally high (>30%), implying this condition acts more as systemic rather than a local disease. This is based on the finding that even in the cases with pelvic nodal and parametrial tumor involvement, the additional presence of malignant cells in peritoneal cytology was associated with decreased survival. 

It remains unknown how the tumor cells of cervical cancer spread into the peritoneal cavity. This study team proposes two possible anatomical pathways for malignant peritoneal cytology: (i) Tumor cells spread through the uterine corpus, adnexa and then the peritoneal cavity, and (ii) tumor cells spread through parametrial tissue, pelvic nodes, followed by a systemic spread including the peritoneal cavity. As the incidence of malignant peritoneal cytology differs across histology types, it is of clinical interest as to whether this is also associated with the infecting HPV subtype. Further study is definitely warranted to prove our hypothesis.

### 4.3. Implication for Postoperative Therapy

Local therapy with pelvic irradiation has been a standard approach to improve survival for high- and intermediate-risk, early-stage cervical cancer [2,3,4]. In our study, malignant peritoneal cytology was associated with decreased survival among those with the high-risk factors, as well as those who received CCRT. In contrast, among those who received systemic chemotherapy, malignant peritoneal cytology was not associated with survival. This suggests that malignant peritoneal cytology is a surrogate of systemic disease spread, and unlike systemic chemotherapy, local therapy may not be adequate to eliminate tumor cells outside of the radiation field. 

Chemotherapy use for malignant peritoneal cytology cases has also been advocated by other researchers [17], however there has been no prior study which has examined the effectiveness of systemic chemotherapy in early-stage cervical cancer with malignant peritoneal cytology.

### 4.4. Strengths and Limitations

Strengths of this study include that it examined the largest sample size in the current body of literature. The analytic approach utilizing PS-IPTW enriched the statistical rigor of the study, and various sensitivity analyses further enhanced the robustness of the results. This is particularly applicable in the analysis of the high-risk group with nodal metastasis or parametrial tumor involvement, as malignant peritoneal cytology is associated with these high-risk factors and the PS-IPTW model showed a medium size effect for these two factors. Finally, the systematic literature review and meta-analysis broaden the interpretation and applicability of this study. 

There are several limitations in this study. First, as is inherent to this type of study, there is unmeasured bias in the analysis. For example, indications and practice patterns for peritoneal cytology testing is unknown. As our study showed that peritoneal cytology testing was commonly performed in higher stage disease, the generalizability of our findings in earlier stages, particularly stage IB1 disease, needs to be examined. Second, central pathology review was not feasible in this study, and thus, the accuracy of malignant peritoneal cytology results was not assessable in the analysis. However, the JGOG-participating sites generally function as referral cancer centers in Japan, and have decent and adequate gynecologic pathology quality care. 

Third, while our study examined a fairly sizable number of cases, the overall number with malignant peritoneal cytology was relatively limited. Therefore, further sensitivity analysis such as a direct comparison between CCRT to systemic chemotherapy within the group of malignant peritoneal cytology was not assessable. Fourth, decision-making for the postoperative therapy choice was not retrievable in the study. Thus, it remains unknown if malignant peritoneal cytology was the exact indication for systemic chemotherapy among those in which it was administered. Fifth, the current study lacks in salient information regarding socio-economic status, a factor which likely impacts the survival of women with cervical cancer. Last, the clinico-pathological characteristics in the malignant peritoneal cytology group were largely different from the negative cytology group, and a small size effect existed even in the weighted model. However, as mentioned above, various sensitivity analyses were deployed to overcome this weakness in the weighted models. 

### 4.5. Clinical Implication 

Current guidelines from multiple organizations do not recommend peritoneal cytology evaluation as a standard practice [2,38]. Based on our results and others, it may be useful to integrate peritoneal cytology testing as a routine surgical procedure at the time of radical hysterectomy for early-stage cervical cancer, as it appears that malignant peritoneal cytology can potentially impact survival. To this end, a further prospective observational study would be necessary to examine this question in early-stage cervical cancer. If malignant peritoneal cytology is indeed found to be prognostic in early-stage cervical cancer, the next logical step would be comparing outcomes with systemic chemotherapy versus CCRT by an interventional approach. Treatment allocation stratified by surgical-pathological factors would be the key, as malignant peritoneal cytology is often associated with aggressive tumor characteristics. Given the rarity of malignant peritoneal cytology in early-stage cervical cancer, national and international collaboration efforts would likely be required.

## 5. Conclusions

The presence of malignant cells in peritoneal cytology may exceed 30% in certain groups of patients with early-stage cervical cancer. Our study suggests that malignant peritoneal cytology may be associated with aggressive tumor characteristics and decreased survival in women with early-stage cervical cancer. The benefit of systemic chemotherapy for this subgroup requires further investigation.

## Figures and Tables

**Table 1:** Patient demographics based on peritoneal cytology performance (*N* = 5942).

Characteristic	Not Performed	Performed	*P*-Value
Number	*n* = 4495	*n* = 1447	
Age	47.6 (±11.9)	49.1 (±12.1)	**<0.001**
Year			0.467 *
2004	825 (18.4%)	239 (16.5%)	
2005	884 (19.7%)	289 (20.0%)	
2006	892 (19.8%)	298 (20.6%)	
2007	957 (21.3%)	335 (23.2%)	
2008	937 (20.8%)	286 (19.8%)	
Clinical stage			**<0.001**
IB1	3,135 (69.7%)	37 (2.6%)	
IB2	395 (8.8%)	501 (34.6%)	
IIA	312 (6.9%)	290 (20.0%)	
IIB	653 (14.5%)	619 (42.8%)	
Histology			0.872
Squamous	2,950 (65.6%)	939 (64.9%)	
Adenocarcinoma	1,118 (24.9%)	361 (24.9%)	
Adenosquamous	367 (8.2%)	128 (8.8%)	
Others	60 (1.3%)	19 (1.3%)	
Surgical volume			**<0.001**
Low	501 (11.1%)	148 (10.2%)	
Mid	2,837 (63.1%)	803 (55.5%)	
High	1157 (25.7%)	496 (34.3%)	
Neoadjuvant chemotherapy			**<0.001**
No	3,869 (86.8%)	969 (67.2%)	
Yes	586 (13.2%)	472 (32.8%)	

Mean (±standard deviation), median (interquartile range), or number (percent per column) is shown. Student *t* test or Fisher exact test, or chi-square test for *P*-Values. Significant *P*-Values are emboldened. Total number may not be 5942, due to missing data. * Cochran-Armitage trend test. Abbreviations: LVSI, lymphovascular space invasion; and CCRT, concurrent chemoradiotherapy.

**Table 2:** Patient demographics based on peritoneal cytology results (*n* = 1409).

Characteristic	No Malignancy	Malignant Cells	*P*-Value	OR (95%CI) ^†^	*P*-Value ^†^
Number	*n* = 1321	*n* = 88			
Age	49.1 (±12.1)	48.8 (±11.6)	0.843		
Year			0.821 *		
2004	218 (16.5%)	16 (18.2%)			
2005	268 (20.3%)	15 (17.0%)			
2006	274 (20.7%)	18 (20.5%)			
2007	298 (22.6%)	26 (29.5%)			
2008	263 (19.9%)	13 (14.8%)			
Clinical stage			**0.039**		
IB1	0	0			
IB2	476 (36.0%)	25 (28.4%)			
IIA	277 (21.0%)	13 (14.8%)			
IIB	568 (43.0%)	50 (56.8%)			
Histology			**<0.001**		<0.001 *
Squamous	902 (68.3%)	28 (31.8%)		1	
Adenocarcinoma	287 (21.7%)	48 (54.5%)		6.18 (3.49-10.9)	**<0.001**
Adenosquamous	117 (8.9%)	9 (10.2%)		2.57 (1.08-6.13)	**0.034**
Others	15 (1.1%)	3 (3.4%)		5.89 (1.25-27.7)	**0.025**
Parametrial involvement			**<0.001**		
No	897 (68.0%)	29 (33.0%)		1	
Yes	423 (32.0%)	59 (67.0%)		1.87 (1.07-3.27)	**0.027**
Node metastasis (pelvic)			**<0.001**		
No	832 (63.6%)	16 (18.4%)		1	
Yes	482 (36.7%)	71 (81.6%)		6.51 (3.43-12.3)	**<0.001**
Node metastasis (para-aortic)			**<0.001**		
No	298 (23.8%)	17 (20.7%)			
Yes	47 (3.8%)	16 (19.5%)			
Not examined *	908 (72.5%)	49 (59.8%)			
Sampled lymph nodes					
Pelvic	39 (IQR 29–51)	36 (IQR 24–51)	0.660		
Para-aortic	10 (IQR 5–17)	14 (IQR 6–29)	0.055		
Deep stromal invasion			**<0.001**		
No	331 (27.3%)	3 (4.1%)			
Yes	881 (72.7%)	71 (95.9%)			
Tumor size			0.736		
≤4 cm	533 (41.5%)	34 (39.1%)			
>4 cm	751 (58.5%)	53 (60.9%)			
LVSI			**<0.001**		
No	403 (32.0%)	7 (8.6%)			
Yes	856 (68.0%)	74 (91.4%)			
Uterine corpus invasion			**<0.001**		
No	999 (78.4%)	35 (42.2%)		1	
Yes	276 (21.6%)	48 (57.8%)		2.74 (1.61-4.67)	**<0.001**
Ovarian metastasis			**<0.001**		
No	1237 (99.0%)	68 (80.0%)		1	
Yes	13 (1.0%)	17 (20.0%)		5.72 (2.34-14.0)	**<0.001**
Surgical volume			**0.033**		
Low	146 (11.1%)	2 (2.3%)			
Mid	726 (55.0%)	54 (61.4%)			
High	449 (34.0%)	32 (36.4%)			
Neoadjuvant chemotherapy			**0.014**		
No	866 (65.8%)	69 (78.4%)			
Yes	450 (34.2%)	19 (21.6%)			
Adjuvant therapy			**0.017**		
None	253 (20.5%)	5 (6.1%)			
Radiotherapy alone	262 (21.2%)	20 (24.4%)			
CCRT	354 (28.6%)	25 (30.5%)			
Chemotherapy alone	348 (28.2%)	29 (35.4%)			
Combined **	19 (1.5%)	3 (3.7%)			

Mean (±standard deviation), median (interquartile range), or number (percent per column) is shown. Student *t* test, Mann-Whitney *U* test, or Fisher exact test, or chi-square test for *P*-Values. Significant *P*-Values are emboldened. Total number may not be 1409, due to missing data. * Cochran-Armitage trend test. ** both systemic chemotherapy and radiotherapy. ^†^ multivariable analysis with a binary logistic regression model (conditional backward): Missing cases were not entered in the model Abbreviations: HR, hazard ratio; CI, confidence interval; LVSI, lymphovascular space invasion; and CCRT, concurrent chemoradiotherapy.

## Supplementary Materials

The following are available online at https://www.mdpi.com/2077-0383/8/11/1822/s1.
