# Supplementary material for: Significance of Malignant Peritoneal Cytology on the Survival of Women with Early-Stage Cervical Cancer: A Japanese Gynecologic Oncology Group Study
Source: J Clin Med. 2019 Nov 1;8(11):1822. doi: 10.3390/jcm8111822 (PMC6953075; doi:10.3390/jcm8111822)
Supplement: Supplementary file 1 [file jcm-08-01822-s001.pdf]

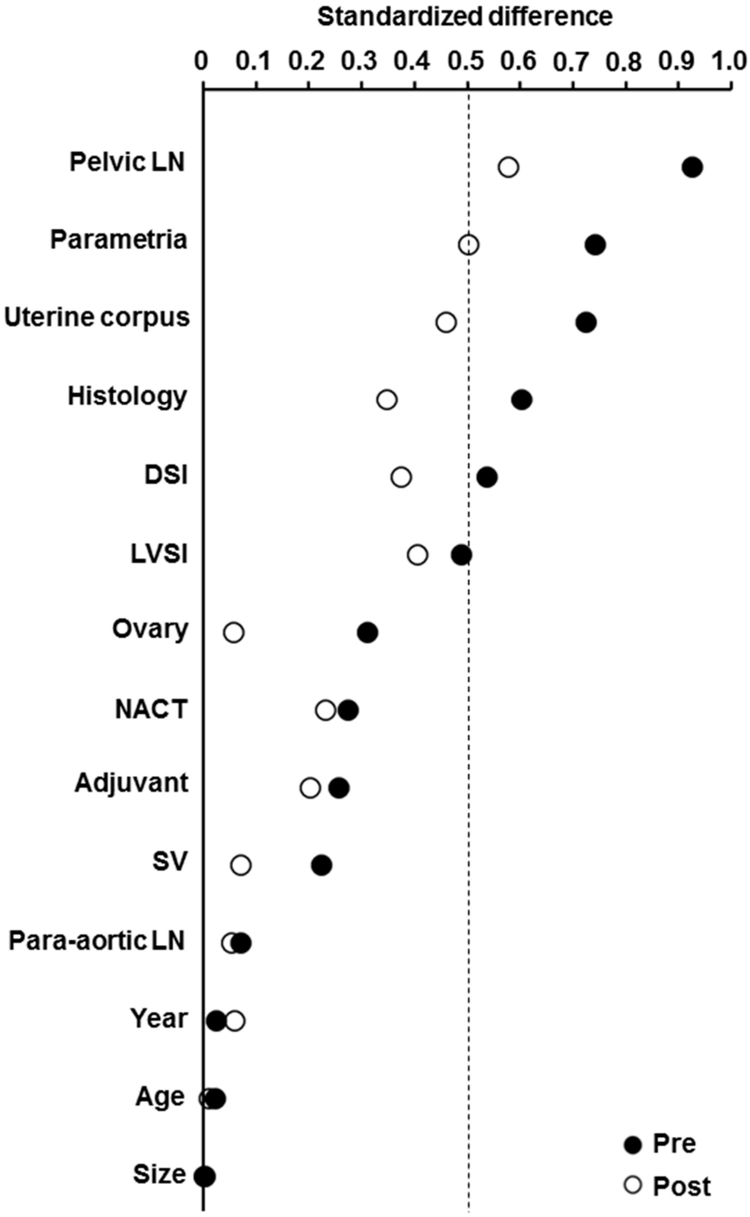

Supplemental Figure S1. Standardized difference before and after PS-IPTW. Standardized differences (SDs) before and after the PS-IPTW are shown. Dash line indicates SD of 0.50, representing the moderate size effect between the two groups. Prior to the PS-IPTW, pelvic lymph node status had large size effect (SD ≥0.80) and parametrial tumor involvement, uterine corpus tumor invasion, histology type, and DSI had medium size effect (SD 0.50-0.79). After the matching, only pelvic lymph node status and parametrial tumor invasion exhibited the medium size effect (SD 0.50-0.79). Abbreviation: LN, lymph node status; DSI, deep stromal invasion; LVSI, lymphovascular space invasion; NACT, neoadjuvant chemotherapy; and SV, surgical volume.

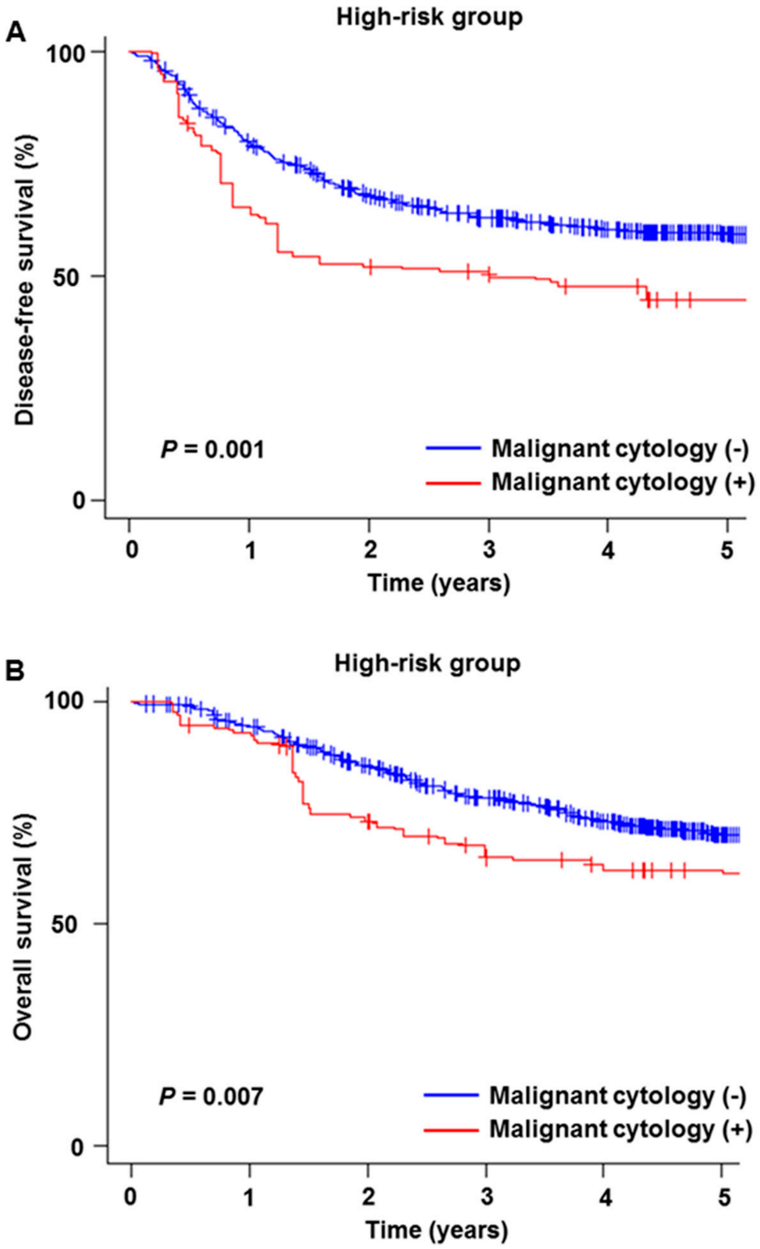

Supplemental Figure S2. Survival curves in the high-risk early-stage cervical cancer (PS-IPTW model). Survival curves based on peritoneal cytology results are shown for (A) disease-free survival and (B) overall survival in the propensity score inverse provability of treatment weighting model.

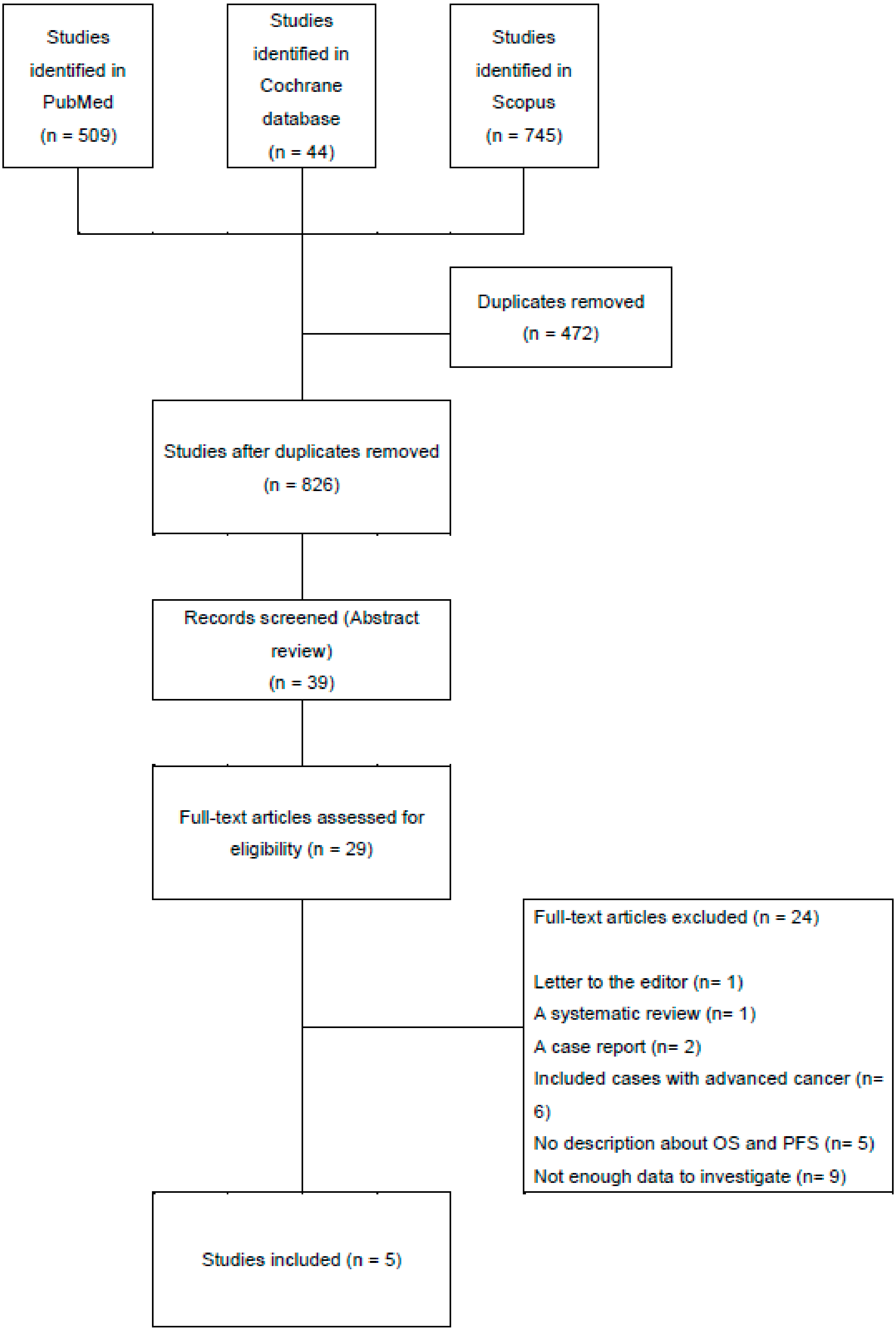

Supplemental Figure S3. Study selection schema for systematic review of literature.

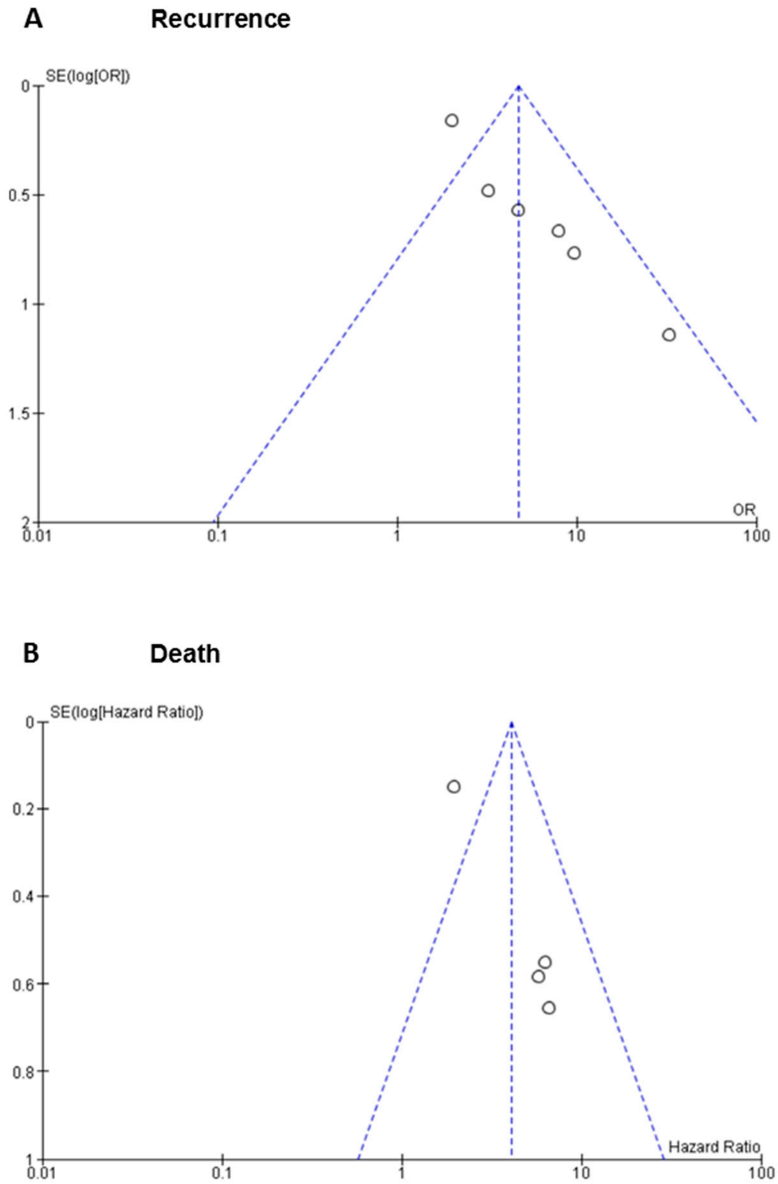

Supplemental Figure S4. Funnel plots for publication bias.
